# A lentiviral sponge for miR-101 regulates RanBP9 expression and amyloid precursor protein metabolism in hippocampal neurons

**DOI:** 10.3389/fncel.2014.00037

**Published:** 2014-02-13

**Authors:** Christian Barbato, Silvia Pezzola, Cinzia Caggiano, Martina Antonelli, Paola Frisone, Maria Teresa Ciotti, Francesca Ruberti

**Affiliations:** Institute of Cell Biology and Neurobiology (IBCN), National Research Council (CNR)Rome, Italy

**Keywords:** Alzheimer's disease, amyloid precursor protein, microRNA, miR-101, Ran-binding protein 9

## Abstract

Neurodegeneration associated with amyloid β (Aβ) peptide accumulation, synaptic loss, and memory impairment are pathophysiological features of Alzheimer's disease (AD). Numerous microRNAs regulate amyloid precursor protein (APP) expression and metabolism. We previously reported that miR-101 is a negative regulator of APP expression in cultured hippocampal neurons. In this study, a search for predicted APP metabolism-associated miR-101 targets led to the identification of a conserved miR-101 binding site within the 3′ untranslated region (UTR) of the mRNA encoding Ran-binding protein 9 (RanBP9). RanBP9 increases APP processing by β-amyloid converting enzyme 1 (BACE1), secretion of soluble APPβ (sAPPβ), and generation of Aβ. MiR-101 significantly reduced reporter gene expression when co-transfected with a RanBP9 3′-UTR reporter construct, while site-directed mutagenesis of the predicted miR-101 target site eliminated the reporter response. To investigate the effect of stable inhibition of miR-101 both *in vitro* and *in vivo*, a microRNA sponge was developed to bind miR-101 and derepress its targets. Four tandem bulged miR-101 responsive elements (REs), located downstream of the enhanced green fluorescence protein (EGFP) open reading frame and driven by the synapsin promoter, were placed in a lentiviral vector to create the pLSyn-miR-101 sponge. Delivery of the sponge to primary hippocampal neurons significantly increased both APP and RanBP9 expression, as well as sAPPβ levels in the conditioned medium. Importantly, silencing of endogenous RanBP9 reduced sAPPβ levels in miR-101 sponge-containing hippocampal cultures, indicating that miR-101 inhibition may increase amyloidogenic processing of APP by RanBP9. Lastly, the impact of miR-101 on its targets was demonstrated *in vivo* by intrahippocampal injection of the pLSyn-miR-101 sponge into C57BL6 mice. This study thus provides the basis for studying the consequences of long-term miR-101 inhibition on the pathology of AD.

## Introduction

Alzheimer's disease (AD) is a progressive neurodegenerative disorder characterized by extracellular senile plaques, intracellular neurofibrillary tangles (Krstic and Knuesel, 2013), and memory loss. Increased amyloidogenic processing of amyloid precursor protein (APP), a type I transmembrane protein, and accumulation of its amyloid β (Aβ) peptide product have important implications for AD pathogenesis. The Aβ peptide is derived from the processing of APP through sequential cleavages by β and γ secretases (Nalivaeva and Turner, 2013). Cleavage by β secretase generates a large soluble fragment sAPPβ and a membrane anchored C-terminal fragment, CTFβ; the latter may be further cleaved by the γ-secretase complex and release Aβ as well as the APP intracellular domain AICD. The Aβ load during disease progression is thought to lead to neurological dysfunction (reviewed in Mucke and Selkoe, 2012).

The APP gene is linked to AD, and familial AD can be caused by increased expression of APP (and consequently Aβ) due to either genomic duplication or regulatory sequence alterations (Podlisny et al., 1987; Rovelet-Lecrux et al., 2006; Theuns et al., 2006).

RanBP9 interacts with APP and influences its trafficking and processing (Lakshmana et al., 2009). RanBP9 is a scaffolding protein involved in the modulation of neuronal functions for the maintenance of cell homeostasis (Suresh et al., 2012). RanBP9 interacts with low-density lipoprotein-related protein (LRP), APP, and BACE1, promoting BACE1-dependent cleavage of APP and Aβ generation both *in vitro* and *in vivo* (Lakshmana et al., 2009, 2012). Furthermore, RanBP9 is increased in mutant APP transgenic mice and in the degenerating brains of patients with AD (Lakshmana et al., 2010, 2012). In addition, RanBP9 overexpression promotes neuronal apoptosis and potentiates Aβ-induced neurotoxicity independently of its capacity to stimulate Aβ generation (Woo et al., 2012), whereas RanBP9 transgenic mice show a significantly increased incidence of synapse loss, neurodegeneration, and spatial memory deficits (Lakshmana et al., 2012; Woo et al., 2012). These various actions of RanBP9 may contribute to the pathogenesis of AD.

The microRNAs represent an emerging class of small non-coding RNA molecules which, in mammals, regulate gene expression primarily by imperfect base pairing with the 3′-untranslated region (UTR) of specific target mRNAs. Hence, microRNAs mediate post-transcriptional repression of target mRNAs (Sun and Lai, 2013). Changes of microRNA expression have been shown to be associated to AD (Tan et al., 2013) and to be deregulated in transgenic animal models of AD (Lee et al., 2012; Barak et al., 2013). MicroRNAs are among the molecules identified as physiological and pathological regulators of key genes involved in AD, including APP (Patel et al., 2008; Hebert et al., 2009; Liu et al., 2010; Vilardo et al., 2010; Long and Lahiri, 2011; Smith et al., 2011; Long et al., 2012; Liang et al., 2012), BACE1 (Hébert et al., 2008; Wang et al., 2008; Boissonneault et al., 2009; Fang et al., 2012; Zhu et al., 2012) and microtubule associated protein tau, MAPT (Hébert et al., 2010). Each microRNA has the potential to target a large number of mRNAs (Friedman et al., 2009) and miRNAs may reinforce their effect through the simultaneous and coherent regulation of multiple targets (reviewed in Inui et al., 2010). Indeed miRNAs that simultaneously regulate APP/BACE1 expression have recently been described (Fang et al., 2012; Ai et al., 2013).

Previously, we demonstrated that the 3′UTR of APP mRNA can functionally interact with miR-101, a brain-enriched microRNA, and that inhibition of endogenous miR-101 increases APP levels. On the other hand, lentivirus-mediated overexpression of miR-101 significantly reduced APP expression and the Aβ load in hippocampal neurons (Vilardo et al., 2010). Furthermore, miR-101 is downregulated in the human AD brain, suggesting that miR-101 expression levels may critically participate in the pathogenesis of AD (Hébert et al., 2008; Nunez-Iglesias et al., 2010).

Here searching for predicted APP metabolism-associated miR-101 targets we further investigated the action of endogenous miR-101 in hippocampal neurons. The hippocampus is affected during the early stages of AD, and changes in the hippocampus coincide with the memory deficits observed in AD patients. Therefore, study of hippocampal cells may improve our understanding of the role of miR-101 and other microRNAs in the gene regulation as well as the involvement of microRNAs in neurodegenerative processes associated with the progression of AD.

Our study provides evidence that miR-101 may regulate not only APP but also RanBP9 in hippocampal neurons both *in vitro* and *in vivo* and that miR-101-mediated post-transcriptional regulation of RanBP9 may modulate the amyloidogenic processing of APP.

## Materials and methods

### Cell culture

Primary hippocampal neurons were prepared from day 17–18 embryos obtained from timed-pregnant Wistar rats (Charles River). Neurons were plated at a density of 1 × 10^6^ cells/dish on 3.5-cm tissue culture dishes pre-coated with poly(D/L-lysine) and cultured in neurobasal medium supplemented with B-27 and GlutaMAX™ (Gibco). Half of the medium was changed every 3–4 days. The neurons were transduced at 7 days *in vitro* with 2 × 10^6^ transducing units (TU)/mL of the pLSyn-miR-101 sponge vector (constructed as described below), or with a control pLSyn vector expressing only EGFP. Protein lysates were collected at 7 days post-transduction and used in Western blotting experiments, as described below.

SH-SY5Y neuroblastoma cells were cultured in Dulbecco's modified Eagle's medium (DMEM) containing 10% fetal bovine serum in a 5% CO_2_-humidified incubator at 37°C. The cells were used in luciferase assays, as described below.

### Luciferase reporter gene constructs and luciferase assay

The SC-RANBP9 3′UTR was purchased from Origene (SC210772). The SC control firefly vector was derived from the SC-RANBP9 3′UTR. The RANBP9 luciferase mutant constructs were generated by using the QuikChange® II Site-Directed Mutagenesis Kit (Stratagene) and the following synthetic oligonucleotides: RanBP9 3′UTR mut up, 5′-ATGGAAGAAATCATTTTTAATGTGTAGAGTAAAACTTGAAATACTCAGGAGCTG-3′; and RanBP9 3′UTR mut down, 5′-CAGCTCCTGAGTATTTCAAGTTTTACTCTACACATTAAAAATGATTTCTTCCAT-3′.

SH-SY5Y cells were plated at a density of 0.6 × 10^5^ per well in 24-well plates and transfected after 24 h with 20 pmole of the indicated microRNA duplexes (Dharmacon) and 50 ng of the firefly luciferase expression vector conjugated to 0.5 μ L Lipofectamine 2000 (Invitrogen) in Opti-MEM® reduced-serum medium (Gibco). Cells were lysed at 24 h after transfection, and luciferase assays were performed by using the Dual Luciferase Reporter Assay System (Promega) according to the manufacturer's protocol. The experiments were carried out in triplicate.

### Preparation of the miR-101 sponge, miRNA and silencing RNA lentiviral vectors, and viral particles

The pLSyn vector was described previously (Barbato et al., 2010). The miR-101 sponge was constructed by using the following synthetic oligonucleotides: S1 CATAATTCAGTTATCCAGTACTGTACGATTTCAGTTATCCAGTACTGTAACCGGT; AS1 TACAGTACTGGATAACTGAAATCGTACAGTACTGGATAACTGAATTATGGTAC; S2 TT CAGTTATCCAGTACTGTATCACTTCAGTTATCCAGTACTGTACCCGGGGGTACCGAGCT; and AS2 CGGTACCCCCGGGTACAGTACTGGATAACTGAAGTGATACAGTACTGGATAACTGAAACCGG. Oligonucleotides S1 and S2, containing four tandem bulged miR-101 binding sites (at position 11), were annealed with AS1 and AS2, respectively, ligated, and digested with the KpnI restriction enzyme. The product was purified and cloned into the KpnI site of the pLSyn backbone, downstream of the woodchuck hepatitis virus post-transcriptional regulatory element. PLB-scr and pLB-101 lentiviral vector have been previously described (Vilardo et al., 2010).

RanBP9 downregulation was induced by using a lentiviral vector expressing a silencing (si) RNA that targets rat RanBP9 mRNA (sh-RanBP9, TRCN0000102112, Sigma-Aldrich), and a control vector expressing a siRNA that does not target rat mRNAs (SHC002, Sigma-Aldrich).

The preparation of the G glycoprotein vesicular stomatitis virus-pseudotyped lentiviral particles has been described previously (Barbato et al., 2010). Briefly, HEK293T cells were transduced with viral vectors, and the virus-containing medium was harvested 60 h later, filtered through a 0.45-mm Durapore Stericup unit, and concentrated by a two-step ultracentrifugation procedure. The titers of the viral vectors used in this study were in the range of 1–3 × 10^9^ TU/ml.

### Animals

A total of 12 males C57BL/6Ncrl mice (Charles River, Italy) were used at the age of 6–7 weeks. All experiments were performed in accordance with European Community Directive 86/609/EC. Mice were unilaterally microinfused into the CA1 field of the hippocampus with lentiviral particles derived from the control pLSyn vector (*n* = 6) or the pLSyn-miR-101 sponge vector (*n* = 6), as described below.

### Intrahippocampal injection of lentiviral vectors

Mice were anesthetized with chloral hydrate (400 mg/kg, i.p.). Holes were drilled above the CA1 field of the hippocampus (anterior/posterior = −2.2, medial/lateral = ±1.8, ventral = −1.8) by using standard stereotaxic procedures. Control pLSyn vector or pLSyn-miR-101 sponge vector (1 μ L) was unilaterally microinfused into the hippocampus via a stainless steel cannula (0.1 mm in diameter) connected to a Hamilton microsyringe. An infusion pump was maintained at an infusion rate of 0.2 μ L/min, and the cannula was left in place for 5 min following completion of the infusion. Two weeks later, the hippocampi were removed and processed for subsequent analyses, as described below.

### RNA extraction and analysis

Total RNA was extracted from primary hippocampal neurons with TRIzol (Invitrogen) according to the manufacturer's instructions. RNA quantitation was performed via quantitative real-time PCR (RT-PCR). The total RNA was treated using the TURBO DNA-free™ Kit (Ambion), reverse-transcribed with SuperScript III reverse transcriptase (Invitrogen), and amplified by using the SensiMixPlus SYBR Kit (BioLine) and the 7900HT Fast Real-Time PCR System (Applied Biosystems). Oligonucleotides amplifying the TATA binding protein (TBP), APP, and RanBP9 were chosen from the Roche Universal Probe Library and were as follows:

rno-TBP-1029-1048FW CCCACCAGCAGTTCAGTAGC
rno-TBP-1081-1103RW CAATTCTGGGTTTGATCATTCTG
rno-APP-FW GCCTGAACTCGAATTAATATACA
rno-APP-RW GCTTCTTCTTCCTCAACATCG
rno-Ranbp9-675-693 FW TGCTTTCACCGACTTACCG
rno-Ranbp9-737-755 RW CCAAAGTTGGCATCAACCA

Relative changes in gene expression were quantified by applying the comparative threshold method (Ct) after determining the Ct values for the reference gene (TBP, the endogenous control) and the target genes in each sample set according to the 2^−ΔΔCt^ method. All reactions were performed in triplicate.

### Protein extraction and western blot analysis

Hippocampal tissues or cultured cells were homogenized in buffer (1% Triton, 0.25% sodium dodecyl sulphate (SDS), 1% sodium deoxycholate, 2 mM EDTA, and 1 mM dithiothreitol) supplemented with a protease inhibitor mixture (Sigma-Aldrich) to yield total protein extracts. The standard 2X Laemmli loading buffer contained 4% SDS, 10% β-mercaptoethanol, 20% glycerol, and 0.04% bromophenol blue in 125 mM Tris-HCl, pH 6.8. Equal amounts of total protein extract were fractionated by electrophoresis in an 8% SDS-polyacrylamide gel and then transferred to a nitrocellulose membrane (Hybond-ECL, GE Healthcare). Membranes were incubated with the indicated primary antibody overnight at 4°C. Incubation with a secondary peroxidase-coupled anti-mouse or anti-rabbit antibody (GE Healthcare) was performed at room temperature for 1 h. Immunoreactivity was determined by using an enhanced chemiluminescence detection kit (Millipore). The following primary antibodies and dilutions were used: mouse monoclonal anti-APP (4G8, 1:500, Signet), rabbit polyclonal anti-sAPPβ (SIG-39138, 1:1000, Covance), rabbit polyclonal anti-RanBP9 (ab78127, 1:2000, Abcam), mouse monoclonal anti-GFP (1:2000, JL-8 632380 Clontech) and anti-glyceraldehyde 3-phosphate dehydrogenase (GAPDH) (1:6000, Covance).

## Results

### RanBP9 is a target of miR-101

To identify putative miR-101 target mRNAs associated with APP metabolism, we used several microRNA target prediction computational programs, such as TargetScan, Pictar, and miRanda (Gomes et al., 2013). Some features of these target prediction programs are: the ability to predict base-pairing patterns and the thermodynamic stability of microRNA-mRNA hybrids, as well as the capacity to perform comparative sequence analysis to evaluate sequence conservation and the individuation of multiple target sites.

**Figure 1:**
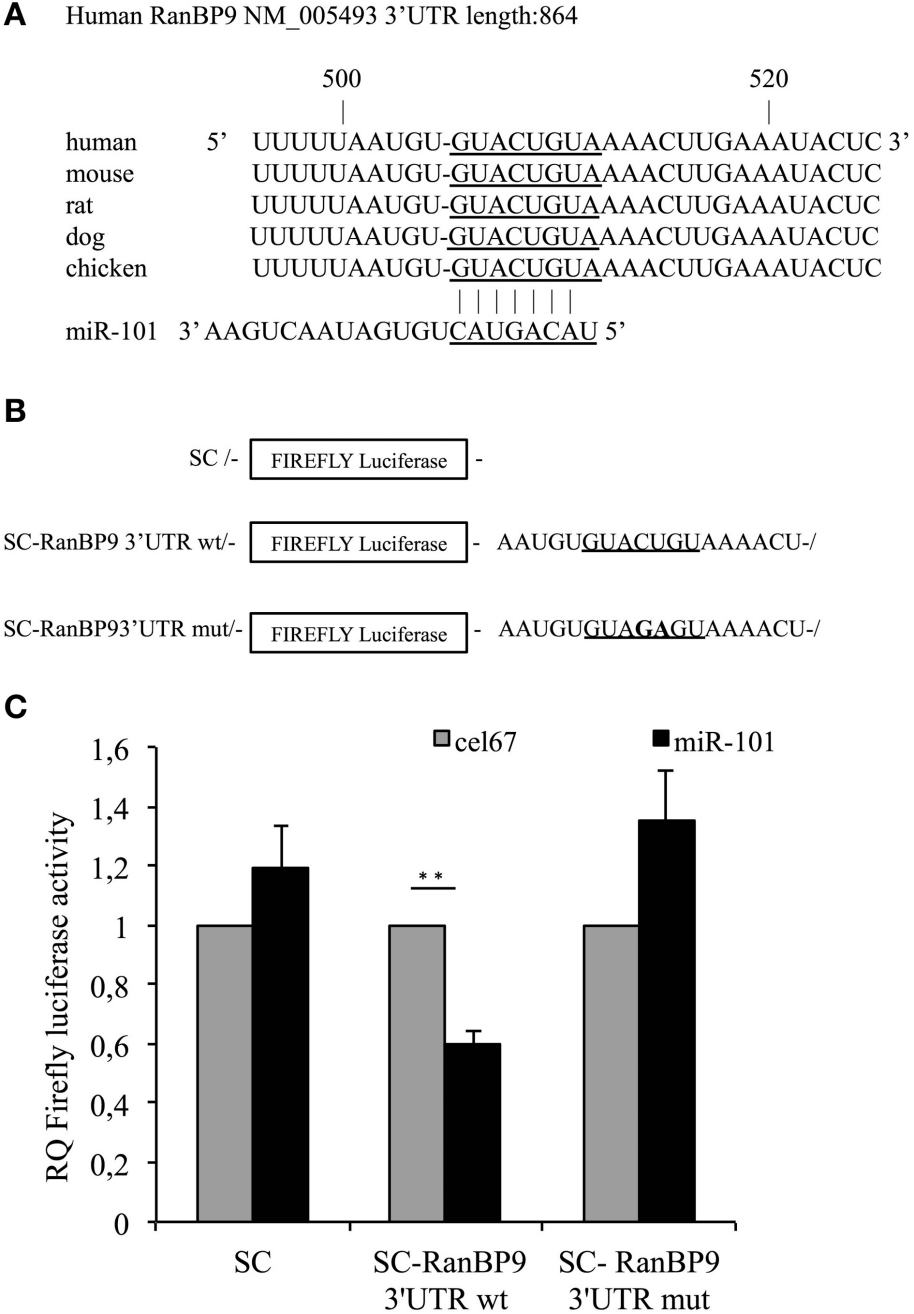
**The conserved miR-101 target site at the 507–513 bp region of the RanBP9 3′UTR is necessary for RanBP9 post-transcriptional regulation. (A)** Ribonucleotide sequences of the conserved putative miR-101 RE within the RanBP9 3′UTR paired with the mature miR-101 sequence. **(B)** Schematic representation of the luciferase constructs used in this study. The miR-101 RE within the RanBP9 3′-UTR is underlined. In the mutant construct, the nucleotides at positions 4 and 5 of the miR-101 RE were mutated, as indicated in bold font. **(C)** SH-SY5Y neuroblastoma cells were transfected independently with the SC control plasmid or with each of two SC-RanBP9 luciferase reporter genes together with either miR-101 or a control microRNA (cel-miR-67) (100 nM). At 24 h post-transfection, luciferase activity was determined. Results are presented as the normalized activity of miR-101-transfected cells relative to that of cells transfected with cel-miR-67. Data are presented as the means ± the SE from three or four independent experiments (^**^p < 0.01, Student's *t*-test).

Our analysis revealed a putative miR-101 RE within the 3′UTR of the mRNA encoding RanBP9 (seed location 507–513 bp), which is conserved among vertebrates (Figure 1A). The RanBP9 protein interacts with the cytoplasmic tails of LRP, APP and BACE1 proteins promoting BACE1-dependent cleavage of APP and Aβ generation both *in vitro* and *in vivo* (Lakshmana et al., 2009, 2012). To determine whether the RanBP9 gene is a target of miR-101, we introduced a firefly reporter vector containing the full-length RanBP9 3′UTR (883 bp) downstream of the luciferase open reading frame into SH-SY5Y neuroblastoma cells. SH-SY5Y cells were co-transfected with either a reporter construct containing the RanBP9 3′UTR or with a control firefly plasmid, in addition to a synthetic miR-101 precursor or a control microRNA (Figures 1B,C). The synthetic miR-101 precursor significantly reduced luciferase expression by more than 40% relative to the control microRNA. The repressive effect of miR-101 on the RanBP9 3′UTR was abrogated by site-directed mutagenesis of the nucleotides at positions 4 and 5 of the miR-101 RE at 507–513 bp. These data indicate that miR-101 negatively regulates RanBP9 expression.

### A lentiviral sponge induces the expression of miR-101 targets in hippocampal neurons

**Figure 2:**
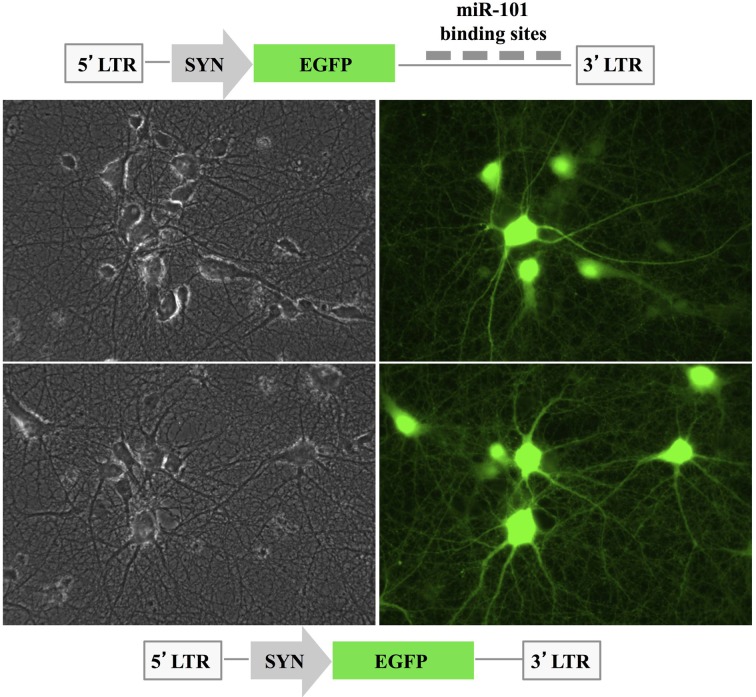
**Expression of the pLSyn-miR-101 sponge in primary hippocampal neurons**. A schematic representation of the pLSyn-miR-101 sponge and the control pLSyn lentiviral vector is shown. The EGFP signal was slightly lower in the pLSyn-miR-101 sponge-containing neurons (top image) at 14 days post-transduction with respect to the pLSyn-containing neurons (bottom image). Left: phase contrast images, right: EGFP signal.

To assay the effects of miR-101 on its target genes and their protein products in hippocampal neurons, we used a lentiviral pLSyn-miR-101 sponge vector in which the synapsin promoter controlled the expression of four tandem bulged miR-101 REs located downstream of the EGFP open reading frame (Figure 2). Rat hippocampal cultures were transduced at 7 days *in vitro* with either the control pLSyn vector or the pLSyn-miR-101 sponge vector. Seven days later, the lentiviral vector-transduced hippocampal neurons expressed EGFP mRNA at a sub-saturating level, resulting in a notable reduction in EGFP fluorescence in the pLSyn-miR-101 sponge-containing neurons vs. the pLSyn vector-containing neurons (Figure 2).

**Figure 3:**
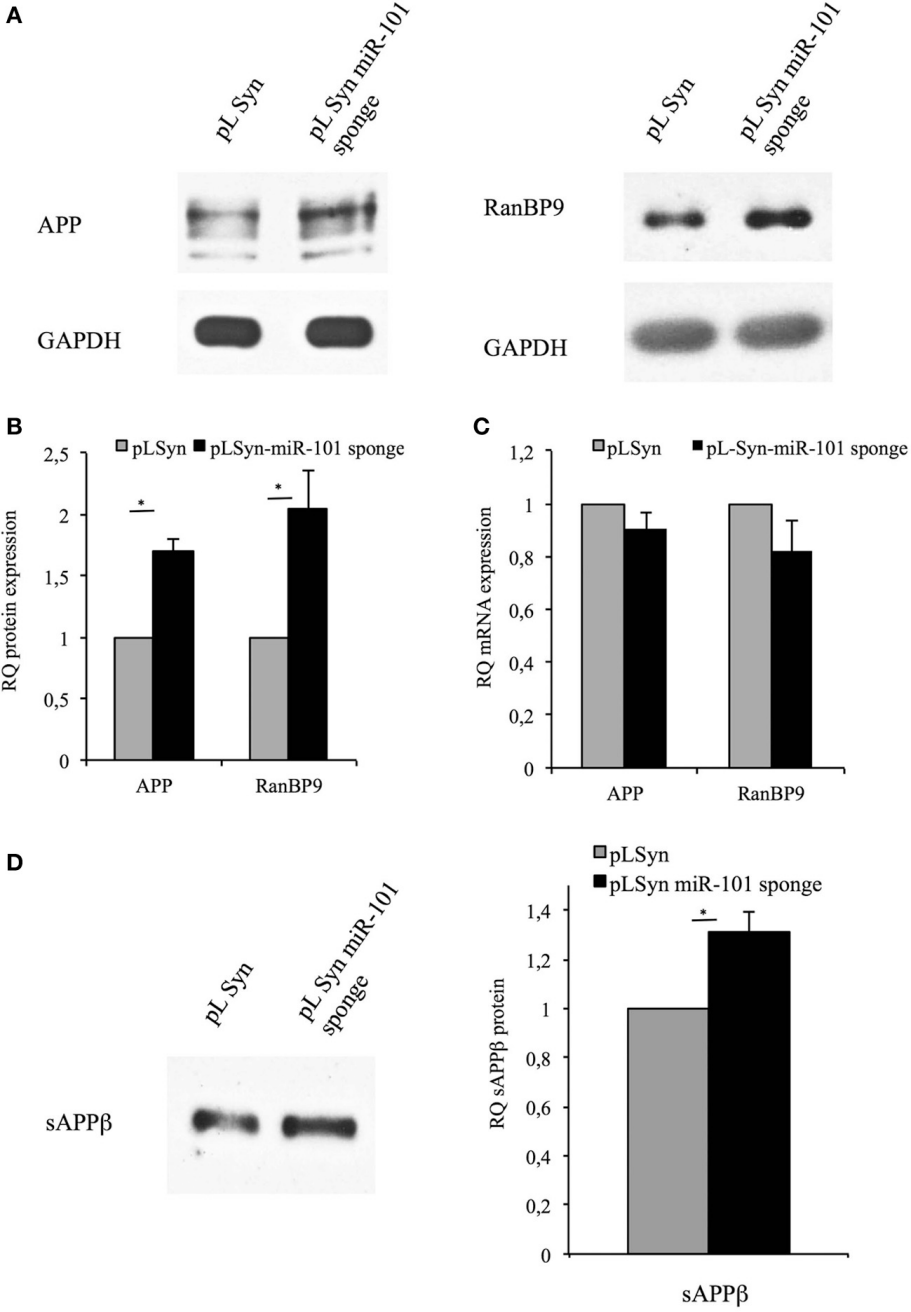
**Inhibition of miR-101 in neurons increases levels of endogenous RanBP9, full-length APP, and its secreted product, sAPPβ**. **(A)** Representative Western blot analysis of protein extracts from lentiviral vector-transduced hippocampal neurons at 7 days post-infection with an antibody recognizing RanBP9, an antibody recognizing full-length APP (4G8), or an antibody recognizing GAPDH. Full blot for RanBP9 with multiple samples is shown in Figure S1. **(B)** The intensities of the bands were quantified by densitometry. The results obtained with the 4G8 antibody or the RanBP9 antibody were normalized to those obtained with the GAPDH antibody and expressed as arbitrary optical density (OD) units. The band intensities for miR-101 sponge-containing neurons were quantified relative to those for control pLSyn vector-containing neurons. **(C)** Quantitative RT-PCR for APP and RanBP9 mRNA, using hippocampal cell total RNA as the template. Expression levels in miR-101 sponge-transduced neurons relative to control pLSyn vector-transduced cells are shown. **(D)** Conditioned culture medium samples from lentivirus-transduced hippocampal neurons were collected from cells cultured in 35-mm dishes, normalized according to the protein content of the corresponding cell extracts, and analyzed by Western blotting with the sAPPβ antibody, which recognizes the secreted form of APP. Cultures expressing the pLSyn-miR-101 sponge exhibited sAPPβ levels that were 1.3-fold higher than the control levels found in pLSyn-transduced cells. Results in **(B–D)** are presented as the means ± the SE of three independent experiments (^*^p < 0.05, Student's *t*-test).

We next evaluated the capacity of the miR-101 sponge to affect miR-101-mediated post-transcriptional regulation by measuring the protein expression levels of APP and RanBP9 in primary hippocampal neurons transduced with either the pLSyn-miR-101 sponge vector or the control pLSyn vector. Western blotting analysis showed that APP and RanBP9 proteins levels were significantly increased in neurons transduced with the pLSyn-miR-101 sponge vector compared with the control pLSyn vector (Figures 3A,B). However, no significant alterations in APP or RANBP9 mRNA levels were observed (Figure 3C). These results suggest that the increase in APP and RanBP9 expression is likely due to a relief of microRNA-mediated translational suppression. Consistently with these data we found that the over-expression of miR-101 in hippocampal neurons induced a significant downregulation of RanBP9 protein (Figure S2).

Because RanBP9 increases the amyloidogenic processing of APP to yield sAPPβ by modulation of BACE1 activity (Lakshmana et al., 2009), sAPPβ levels in the extracellular medium of lentiviral vector-transduced hippocampal neurons were next examined. The secretion of sAPPβ into the conditioned culture medium was significantly increased for neurons transduced with the pLSyn-miR-101 sponge vector relative to the control pLSyn vector (Figure 3D). This result demonstrates that the inhibition of miR-101 action on its target genes increases the amyloidogenic processing of APP.

### Silencing of RanBP9 after miR-101 inhibition mitigates sAPPβ overproduction

**Figure 4:**
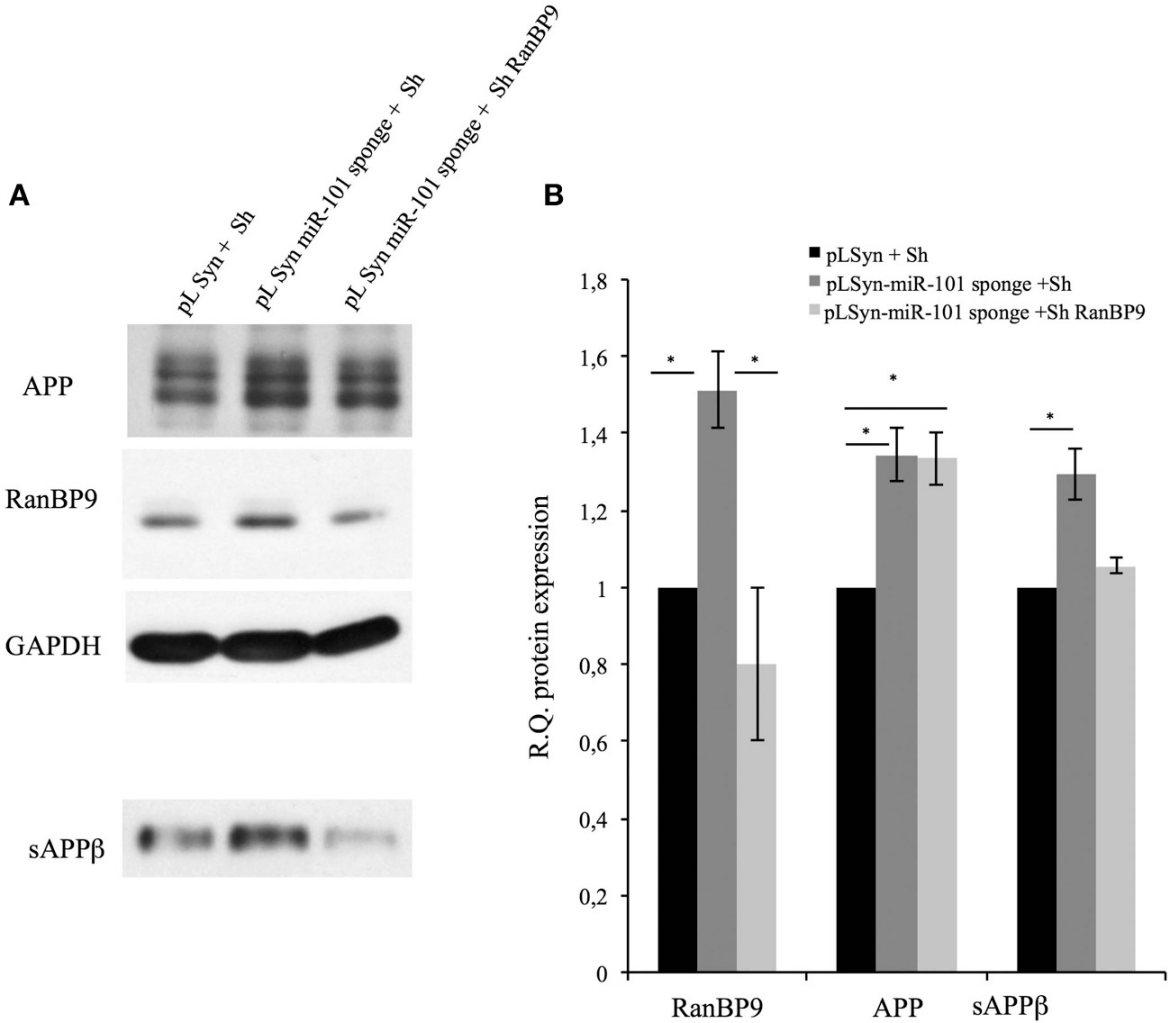
**Silencing of RanBP9 reverses oversecretion of sAPPβ into the conditioned medium of pLSyn-miR-101-containing hippocampal neurons**. Hippocampal neurons at 7 days *in vitro* were infected with the control pLSyn vector or the pLSyn-miR-101 sponge vector. Seventy-two hours later, the cells were transduced with a lentiviral vector containing RanBP9 siRNA under the control of the U6 promoter, or with a control lentivirus expressing a scrambled siRNA. After 14 days, the neurons were collected for Western blot analysis. **(A)** Representative immunoblots of protein extracts from lentiviral vector-transduced neurons reacted with an antibody recognizing RanBP9, an antibody recognizing full-length APP (4G8), or an antibody recognizing GAPDH are shown. Alternatively, conditioned culture medium samples were collected from lentiviral vector-transduced hippocampal neurons cultured in 35-mm dishes, normalized according to the protein content of the corresponding cell extracts, and analyzed by Western blotting with the sAPPβ antibody. **(B)** Quantitative data are provided in the histogram. The data are expressed as means ± the SE of three independent experiments (^*^p < 0.05, Student's *t*-test).

To evaluate the role of RanBP9 in sAPPβ overproduction, we analyzed the levels of sAPPβ in pLSyn-miR-101 sponge-containing hippocampal cultures in which RanBP9 was downregulated. Cultured hippocampal neurons expressing the miR-101 sponge were transduced with a lentiviral vector containing RanBP9 siRNA under the control of the U6 promoter, or with a control lentiviral vector expressing a scrambled siRNA. The pLSyn vector-containing hippocampal neurons transduced with the scrambled lentiviral vector were used as the experimental control. Neurons were collected at 96 h after RanBP9 silencing and subjected to Western blot analysis for APP, RanBP9, and sAPPβ (Figure 4). The pLSyn-miR-101 sponge-containing neurons transduced with the scrambled siRNA showed an increase in all three proteins compared with the control neurons. On the other hand, the pLSyn-miR-101 sponge-containing neurons with silenced RanBP9 showed a positive correlation between decreased RanBP9 expression and attenuated sAPPβ secretion into the conditioned medium (Figure 4). This observation suggests that RanBP9 upregulation may increase sAPPβ production when miR-101 is inhibited.

### The miR-101 sponge induces APP and RanBP9 expression *in vivo*

**Figure 5:**
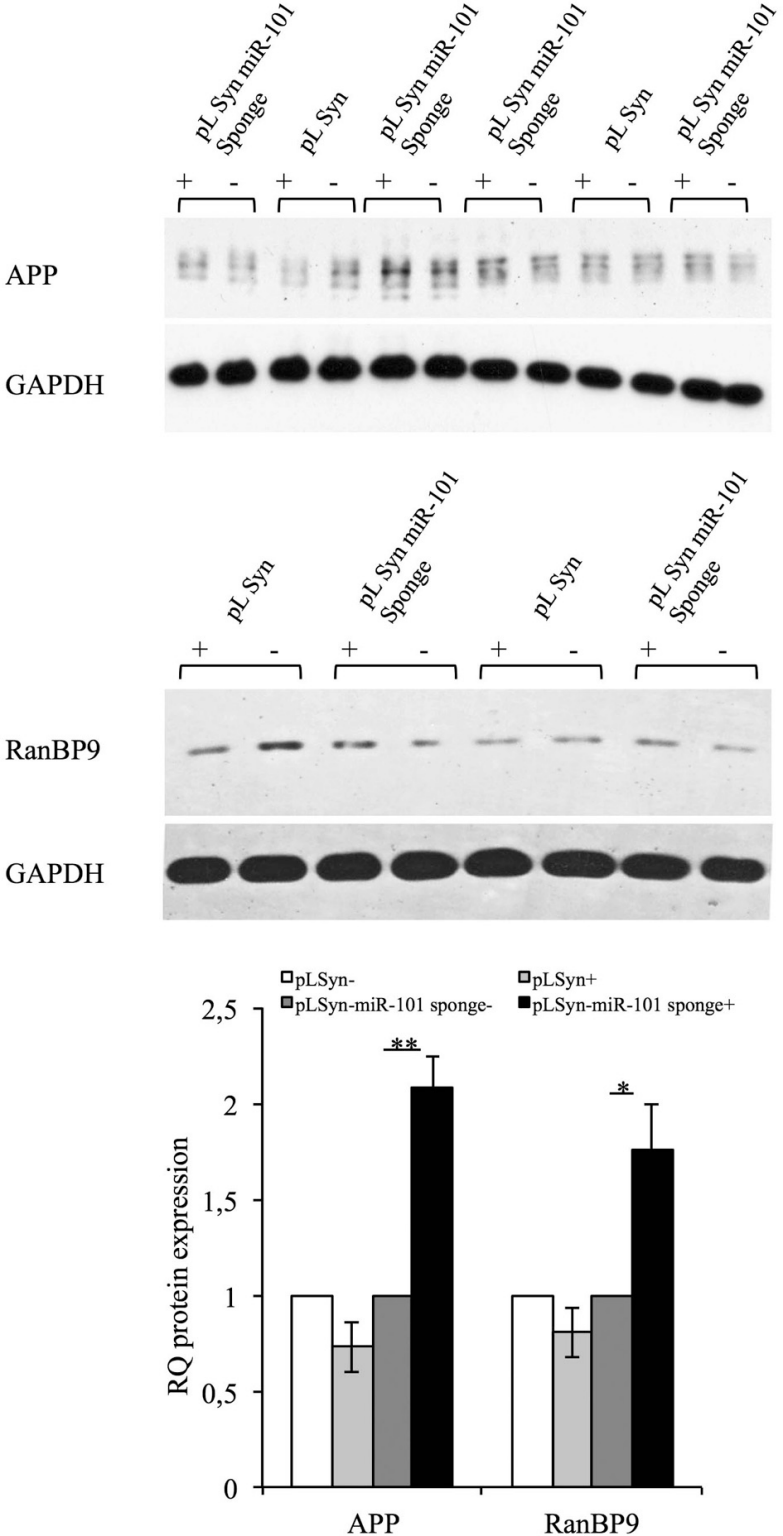
**Full-length APP and RanBP9 are targets of miR-101 in mouse hippocampal neurons *in vivo***. Western blotting was performed of hippocampal tissues injected and non-injected with lentiviral vectors. Effective expression of the lentiviral vector was revealed by the presence of EGFP (data not shown). Expression of the miR-101 lentiviral sponge resulted in an increase in the protein expression levels of the miR-101 targets, APP and RanBP9, with respect to non-injected tissue. APP and RanBP9 band intensities were quantified by densitometry, normalized to the GAPDH signal, and expressed as arbitrary OD units. The relative ratio of APP (pLSyn *n* = 3; pLSyn-miR-101 sponge *n* = 4) and RanBP9 (pLSyn *n* = 3; pLSyn-miR-101 sponge *n* = 4) expression in injected vs. non-injected tissue is shown. Results are presented as the means ± the SE (^*^p < 0.05; ^**^p < 0.01, Student's *t*-test).

The significance of miR-101-mediated post-transcriptional regulation on the expression of its targets was evaluated *in vivo* by unilaterally injecting lentiviral particles into the hippocampus of adult mice. Infection was monitored by western blot analysis of EGFP expression. Two weeks after injection, the right and left sides of the hippocampus were processed for biochemical analysis. Western blotting showed that the expression of the miR-101 sponge induced the upregulation of the APP and RanBP9 proteins in the injected side of the hippocampus relative to the non-injected side (Figure 5). The injection of the control lentivirus did not appreciably alter the expression of either miR-101 target (Figure 5). These findings indicate that miR-101-mediated post-transcriptional regulation is involved in the APP metabolism of adult neurons both *in vivo* and *in vitro*.

## Discussion

MicroRNAs are abundantly expressed in the brain, where they play important roles in neural development and function. MicroRNAs also feature predominantly in gene regulation under both normal and pathological conditions (Eacker et al., 2013). Changes in microRNA expression are found in the brains of patients affected by various neurological diseases, including AD. Several *in vitro* and *in vivo* studies explored the functional role of microRNAs in AD pathogenesis, and showed that these molecules are potentially involved in the regulation of APP metabolism (reviewed in Delay et al., 2012). Notably, aberrant processing of APP is implicated as a causative factor in AD (Nalivaeva and Turner, 2013).

Recently, we reported that miR-101 is among the microRNA species that target the APP gene (Vilardo et al., 2010). In addition, miR-101 is also downregulated in the human AD cerebral cortex (Hébert et al., 2008; Nunez-Iglesias et al., 2010). Here, we demonstrate that the RanBP9 gene is a novel target of miR-101, and that miR-101 is involved in the amyloidogenic processing of APP by downregulating the expression of the RanBP9 protein. RanBP9 regulates Aβ peptide production in several cell lines and primary neuronal cultures and exerts its actions by forming protein complexes with APP, LRP, and BACE1, leading to the increased proteolytic processing of APP, secretion of sAPPβ, and generation of Aβ, which is present in excessive amounts in the brains of AD patients. Through the use of computational prediction programs, we found a putative conserved miR-101 RE within the RanBP9 mRNA 3′UTR. Furthermore, luciferase assays and site-directed mutagenesis experiments demonstrated a functional interaction between the RanBP9 3′UTR and miR-101.

It is worth mentioning that beyond miR-101, among several microRNAs reported to regulate APP expression, miR-153 (Long et al., 2012) is also predicted to target human and mouse, but not rat, RanBP9 3′UTR. However, the miR-153 binding site is very close to the RanBP9 open reading frame suggesting that the efficiency of targeting should be low (Grimson et al., 2007).

Next, we demonstrated the specific relevance of miR-101-mediated post-transcriptional regulation of the RanBP9 gene to APP metabolism in hippocampal neurons. We first took advantage of the neuron-selective synapsin promoter (Kügler et al., 2003) to express a lentiviral miR-101 sponge in cultured neurons. This sponge interferes with the actions of miR-101 on its targets, including the RanBP9 gene. Inhibition of miR-101 via delivery of the sponge increased protein expression levels of APP and RanBP9, as well as the secretion of sAPPβ. Moreover, the overproduction of sAPPβ in the extracellular medium of pLSyn-miR-101 sponge-containing neurons was reversed by RanBP9 silencing. Intrahippocampal injection of lentiviral sponge particles in adult mice also demonstrated that the APP and RanBP9 genes are regulated by miR-101 in adult hippocampal neurons *in vivo*.

Our data indicate that RanBP9 translation may be modulated by microRNA post-transcriptional regulation. On the other hand a recent study suggests that additional pathways may regulate RanBP9 protein expression (Wang et al., 2013). Indeed RanBP9 protein half-life is increased by COPS5, a novel RanBP9-interacting protein that increases the stability of its interacting proteins most likely through an action on the ubiquitin-proteosome system (Wang et al., 2013).

APP expression is extensively regulated at post-transcriptional level by several microRNAs and different RNA binding proteins (reviewed by Ruberti et al., 2010). To date, however, interactions between these regulatory mechanisms on APP expression have not been evidenced. The Fragile X Mental Retardation Protein (FMRP) binds APP mRNA and represses its translation (Westmark and Malter, 2007). Interestingly RanBP9 may interact with FMRP and inhibit its RNA binding activity (Menon et al., 2004). It would therefore be tempting to speculate that APP protein levels may be influenced by miR-101 post-transcriptional regulation of FMRP/RanBP9 complex. Since RANBP9 silencing in pLSyn-miR-101 sponge neurons did not reverse APP protein overexpression we can exclude that RANBP9 protein levels were indirectly modulating APP translation.

Our data showed that miR-101 regulates two target genes that are closely associated with AD, Further investigations are required to determine whether alteration of this regulatory mechanism may affect AD pathogenesis. Importantly, experimental evidence suggests that if the amyloidogenic processing of APP could be halted or decelerated, the devastating effects of AD might be mitigated. Thus, the elucidation of which microRNAs act on APP metabolism provides researchers with a specific focus for the development of new drugs for the management of AD. Furthermore, the demonstration that RanBP9 is post-transcriptionally regulated by miR-101 might have interesting disease implications independently of its effects on amyloidogenic processing, because the overexpression of RanBP9 can induce neuronal apoptosis without generating Aβ peptides (Woo et al., 2012), and RanBP9 transgenic mice show synapse loss, neurodegeneration, and spatial memory deficits (Lakshmana et al., 2012; Woo et al., 2012).

Finally, miR-101 is downregulated in the hippocampus of very old vs. young wild-type mice, and in both very old and young mouse models of AD compared with their age-matched normal controls (Barak et al., 2013). Further studies in mouse models may therefore elucidate the consequences of long-term inhibition of miR-101 on AD pathology.

### Conflict of interest statement

The authors declare that the research was conducted in the absence of any commercial or financial relationships that could be construed as a potential conflict of interest.

## Supplementary material

Figure S1 **The gel blot of RanBP9 protein level in three independent hippocampal cultured neurons expressing the miR-101 sponge construct (pLSyn-miR-101 sponge) and the parental control vector (pLSyn)**. Two exposure time of RanBP9 are shown.

Figure S2 **Reduction of RanBP9 protein levels by miR-101 overexpression. (A)** Western immunoblots show the expression of RanBP9 and APP in hippocampal neurons that were transduced with either a control lentiviral vector (pLB-scr) expressing a control microRNA or a lentiviral vector expressing a mature miR-101 (pLB-101) **(B)** GAPDH-relative expressions are shown in the histogram. Data are presented as the means ± the *SD* (^*^*p* < 0.05; ^**^*p* < 0.01, Student's *t*-test; *n* = 3).
