# Supplementary figures and images for: A lentiviral sponge for miR-101 regulates RanBP9 expression and amyloid precursor protein metabolism in hippocampal neurons
Source: Front Cell Neurosci. 2014 Feb 13;8:37. doi: 10.3389/fncel.2014.00037 (PMC3923151; doi:10.3389/fncel.2014.00037)

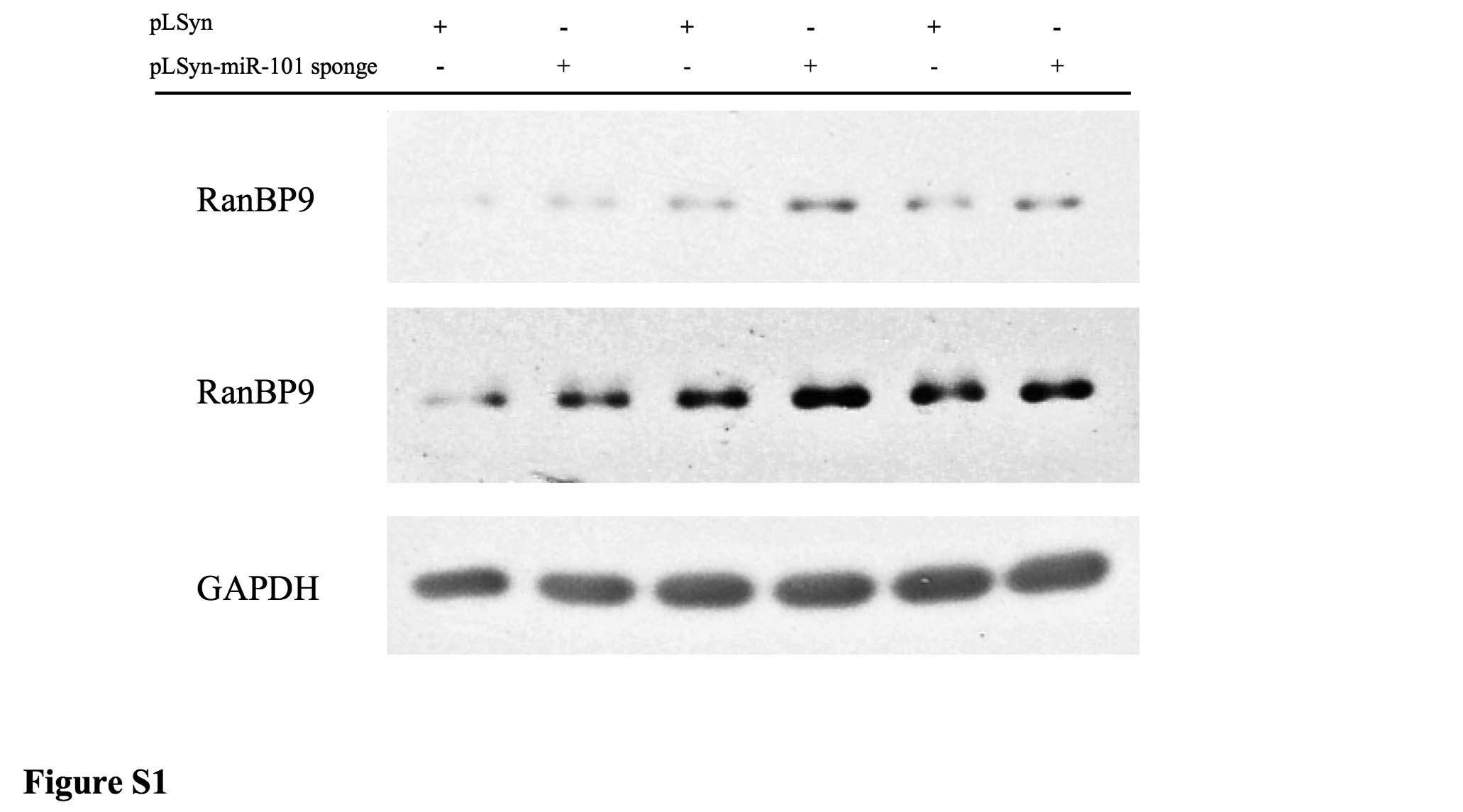

Supplement: Figure S1 — The gel blot of RanBP9 protein level in three independent hippocampal cultured neurons expressing the miR-101 sponge construct (pLSyn-miR-101 sponge) and the parental control vector (pLSyn). Two exposure time of RanBP9 are shown. [file Presentation1.ZIP › 70543_Ruberti_Suppl Figure_1.JPEG]

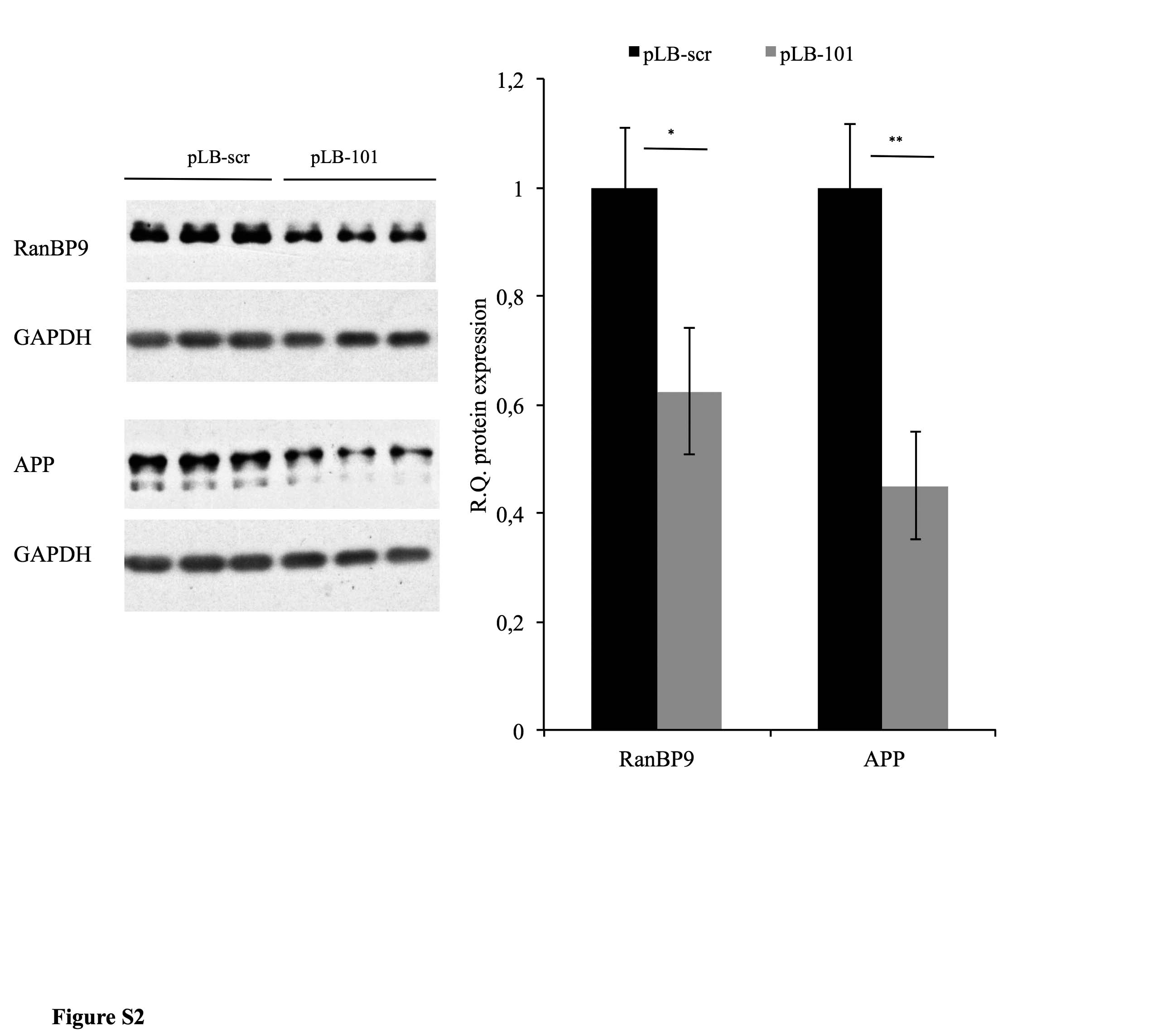

Supplement: Figure S1 — The gel blot of RanBP9 protein level in three independent hippocampal cultured neurons expressing the miR-101 sponge construct (pLSyn-miR-101 sponge) and the parental control vector (pLSyn). Two exposure time of RanBP9 are shown. [file Presentation1.ZIP › 70543_Ruberti_Suppl Figure_2.JPEG]
